# Relative Undernourishment and Food Insecurity Associations with *Plasmodium falciparum* Among Batwa Pygmies in Uganda: Evidence from a Cross-Sectional Survey

**DOI:** 10.4269/ajtmh.13-0422

**Published:** 2014-07-02

**Authors:** Joseph A. Lewnard, Lea Berrang-Ford, Shuaib Lwasa, Didacus Bambaiha Namanya, Kaitlin A. Patterson, Blánaid Donnelly, Manisha A. Kulkarni, Sherilee L. Harper, Nicholas H. Ogden, Cesar P. Carcamo, the Indigenous Health Adaptation to Climate Change Research Team 2013

**Affiliations:** Department of Geography, McGill University, Montreal, Quebec, Canada; Department of Geography, Makerere University, Kampala, Uganda; Ugandan Ministry of Health, Kampala, Uganda; Department of Epidemiology and Community Medicine, University of Ottawa, Ottawa, Ontario, Canada; Department of Population Medicine, Ontario Veterinary College, University of Guelph, Guelph, Ontario, Canada; Zoonoses Division, Public Health Agency of Canada, Saint-Hyacinthe, Quebec, Canada; School of Public Health and Administration, Universidad Peruana Cayetano Heredia, Lima, Peru

## Abstract

Although malnutrition and malaria co-occur among individuals and populations globally, effects of nutritional status on risk for parasitemia and clinical illness remain poorly understood. We investigated associations between *Plasmodium falciparum* infection, nutrition, and food security in a cross-sectional survey of 365 Batwa pygmies in Kanungu District, Uganda in January of 2013. We identified 4.1% parasite prevalence among individuals over 5 years old. Severe food insecurity was associated with increased risk for positive rapid immunochromatographic test outcome (adjusted relative risk [ARR] = 13.09; 95% confidence interval [95% CI] = 2.23–76.79). High age/sex-adjusted mid-upper arm circumference was associated with decreased risk for positive test among individuals who were not severely food-insecure (ARR = 0.37; 95% CI = 0.19–0.69). Within Batwa pygmy communities, where malnutrition and food insecurity are common, individuals who are particularly undernourished or severely food-insecure may have elevated risk for *P. falciparum* parasitemia. This finding may motivate integrated control of malaria and malnutrition in low-transmission settings.

## Introduction

*Plasmodium falciparum* malaria and malnutrition are co-occurring health risks within populations globally; however, nutritional modulation of individual risk for *P. falciparum* parasitemia is complex and poorly understood.^1^ Although poverty and host-level biological pathways contribute to the dual burden of malnutrition and malaria within resource-poor populations, relative contributions of such distal and proximate factors remain unclear.^1^–^4^ Low-transmission tropical areas at high altitudes, including the east African highlands, are forecasted to account for a large share of malaria resurgence under projected changes in temperature and precipitation regimes associated with global climatic change.^5^–^12^ Because vector exposure is heterogeneous in these settings and prevalence of naturally acquired immunity tends to be low in resident populations,^13^ host-level behavioral and immunological factors are of particular importance to individual risk for infection.^14^–^18^ However, low- and variable-transmission settings are underrepresented within present literature relating malaria risk to nutrition. We thus lack an evidence base to inform integrated management of malaria and nutrition in areas at risk for becoming hyper- or holoendemic as climate change alters vector habitat suitability.

Presently hypothesized associations between malaria risk and nutrition suggest that resolving a population's chronic and acute nutrient deficiencies may lead to any of several scenarios for malaria control. Generally, improved individual immune function with better nutrition may limit health burdens from malaria resurgence^3^,^19^–^21^ by reducing morbidity and mortality associated with parasitemia.^22^ Alternatively, host-level protective effects of iron and folic acid deficiencies against *Plasmodia* may indicate that micronutrient supplementation could increase incidence of clinical malaria within an endemic population^23^–^26^ and heighten severity of disease episodes.^3^,^4^,^26^–^31^ In the event that such antagonistic effects are self-canceling,^32^ reducing prevalence of malnutrition may serve more distally as a poverty-reduction tool to facilitate malaria control (for instance, by alleviating economic and life-year losses from incapacitation).^33^–^36^ Observational evidence for all three scenarios indicates demand for better understanding of factors driving local variation in how malnutrition and malaria relate epidemiologically and interventions tailored to local circumstances, which may differ with respect to transmission intensity, prevalence of acquired immunity, and nutritional or food security risks.

Household food insecurity, defined here as constrained social, economic, and physical access to sufficient and nutritionally adequate foods for maintaining a healthy and active lifestyle, is a determinant for malnutrition and associated comorbid conditions globally^37^,^38^ and specifically, within sub-Saharan Africa.^39^ Although food insecurity is often a perennial state, it may additionally occur intermittently when conditions surrounding food production or acquisition become adverse.^40^–^42^ Because individuals can be inclined to reduce dietary quality or variety as a coping mechanism in such events,^37^,^43^ transitory food insecurity may cause acute micronutrient deficiency, even in individuals who are typically well-nourished.^44^–^46^

Relationships among food insecurity, malnutrition, and risk for malaria have been sparsely investigated and are difficult to observe given the multifactorial host-level biology of malnutrition and immune function, the need of *P. falciparum* for labile micronutrients within a human host, reverse-causal effects of malarial anemia on anthropometric indicators for nutritional status, and external socioenvironmental factors predisposing individuals to risk for all three conditions. For instance, as a cause for poor mental health outcomes, including psychological stress, food insecurity may disrupt normal host endocrine function and increase susceptibility to *P. falciparum* or other infections.^47^–^51^ More proximately, food insecurity serves as a determinant for malnutrition, which may hamper immune responses to *Plasmodium* infection while also limiting nutrient availability for parasite reproduction and pathogenesis within the human host.^31^ Heterogeneous prevalence of acquired immunity among settings with different transmission patterns may likewise contribute to variation in observed nutrition and food security associations with risk for parasitemia or clinical illness.^13^ Of the two contemporary studies assessing food insecurity associations with clinical malaria, one study conducted within Haiti identified food insecurity as a risk factor among children under 5 years old,^19^ whereas one study in Colombia identified no significant association among children under 15 years old.^52^ Both regions have low transmission, respectively, of *P. falciparum* and *P. vivax*, with parasite prevalence between 3% and 4% and high adult to children under 5 years case ratios (> 1) characteristic of unstable malaria epidemiology.^1^,^53^–^55^ Presently, no studies have assessed food security and risk for parasitemia.

As a marginalized, semisubsistent Indigenous population with pre-existing burden from these climate-sensitive health risks, the Batwa pygmies of Uganda are expected to be vulnerable to increasing malaria, food insecurity, and malnutrition amidst climate change.^56^–^59^ Together with monetary poverty, coerced migration from their ancestral forest home to agrarian settlements has constrained the Batwa's coping options during frequent episodes of famine and has not reduced health gaps relative to other Ugandans.^56^ Average life expectancy at birth is only 28 years compared with the Ugandan average of 53 years; the Batwa child mortality ratio of 41% is high relative to the southwestern Ugandan average of 18.1% and the national average of 13.7%.^56^ Although we know that *P. falciparum* transmission is generally low and volatile in Uganda's southwestern highlands,^60^,^61^ prevalence and causes for individual infection among the Batwa, whose settlements are geographically separated from the settlements of the ethnic majority population, have not been assessed.

Here, we present the outcomes of a malariometic, anthropometric, and food security census among Batwa pygmies residing in Kanungu District, Uganda. Our objectives were to (1) characterize the distribution of *P. falciparum* infection among Batwa pygmies and (2) contribute observational evidence of whether risk for current *P. falciparum* infection covaries with food insecurity or nutritional status in a low-transmission setting.

## Materials and Methods

### Ethics.

The study was approved in accordance with the requirements of the McGill University Policy on the Ethical Conduct of Research Involving Human Subjects and the Canadian Tri-Council Policy Statement on Ethical Conduct for Research Involving Human Subjects; the latter affords particular attention to issues among Indigenous and disadvantaged peoples. We, moreover, followed best practice frameworks for working with Indigenous communities in climate change vulnerability research.^59^,^62^,^63^ We obtained informed consent from adult participants for each component of the clinical, household, and anthropometric surveys and from parents for children under 18 years old. Parents or guardians answered on behalf of children under 12 years old.

### Design.

**Figure 1.**
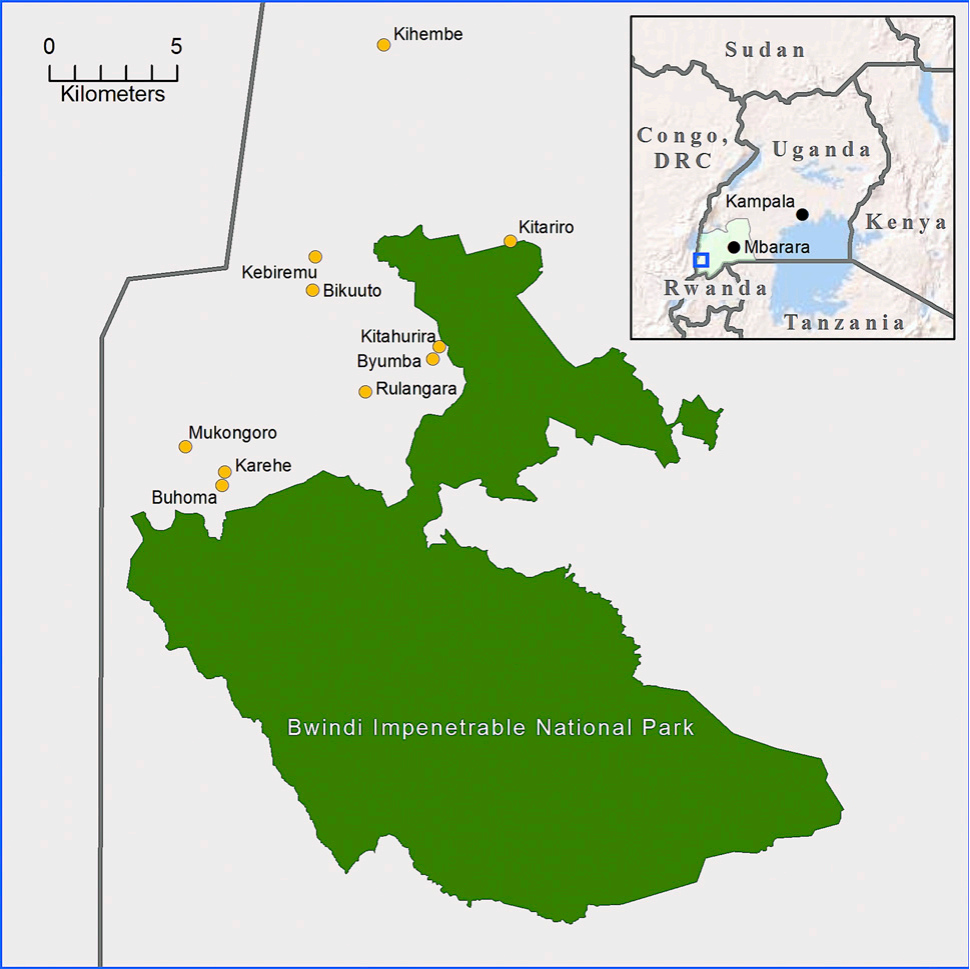
Map of the study region. The locations of 10 Batwa settlements within Kanungu District, Western Region, Uganda (Inset) are shown. The study communities are situated between the Bwindi Impenetrable National Park and the border with the Democratic Republic of the Congo (DRC).

During the biannual transmission peak from January to February of 2013,^60^ we administered a standardized questionnaire addressing issues in environment and health identified by Batwa communities during a pilot research phase.^56^ We pre-tested the survey instrument during reduced seasonal administrations preceding the January–February 2013 census. We obtained finger-prick blood samples from all consenting participants for in-field rapid *P. falciparum* diagnostic tests. Respondents were interviewed in the Rukiga language by paid interviewers from Kanungu District. We sought to achieve census-level representation across all ages within Kanungu District's 10 Batwa settlements (mapped in Figure 1) as identified by partners from the Batwa Development Program and Bwindi Community Hospital.^64^ Children who attended residential school were in their home communities during the surveying because of the Ugandan school holiday, maximizing survey response. We constructed a sampling frame in field by verifying with village chiefs the number and composition of households within their communities. We asked household heads to identify co-occupants and if co-occupants were not present at the interview, indicate their whereabouts. We visited individuals who did not report to interview sites at their homes and worksites.

### Food security assessment.

We assessed food insecurity using a modified Rukiga-language translation of the US Department of Agriculture (USDA) Food Security Supplement to the Current Population Survey (CPS).^65^ We used a 3-month recall period to understand participants' access to food during the preceding rainy season. Adapting the CPS survey as a metric for international settings is common practice and has been successful in developed as well as developing countries.^66^,^67^ Outcomes comprised an ordinal scale, including four levels ranging from severe food insecurity to food security.

### Anthropometric assessment.

We assessed variation in acute nutritional status using individuals' relative standing within the observed distribution of age- and sex-specific mid-upper arm circumference (MUAC), which was measured on an individual's left arm halfway between the tip of the shoulder and the tip of the elbow. In undernourished populations, MUAC outperforms body mass index (BMI) as an anthropometric measure for acute nutritional status among children and adults and as a prognostic indicator for malnutrition-related outcomes.^68^ It has been used frequently within pygmy populations,^69^–^71^ among whom BMI and its prognostic indications have not been validated. Our analysis used relative MUAC within the study population, a technique validated in prior work with children and adults,^72^–^75^ because there is debate concerning optimal MUAC cutoff points for signifying acute malnutrition, how such cutoffs differ between women and men,^76^–^80^ and the fact that there is no evidence that non-pygmy–derived MUAC cutoffs are valid within pygmy populations. For age- and sex adjustment, we calculated mean MUAC and SDs within 3-year age- and sex-specific groupings between ages 6 and 17 years and among individuals of age 18+ years. We centered MUAC measurements on mean for age–sex class and scaled by SD within each class to produce normal (*z*) scores for the analysis.

### Rapid diagnostic testing.

Parasitemia was inferred from positive outcome to an SD-BIOLINE rapid immunochromatographic assay detecting Histidine-rich protein II (HRP-II) antigen from *P. falciparum* (Standard Diagnostics, Inc., Yongin-si, Gyeonggi-do, Republic of Korea). The test additionally bound *Plasmodium* lactate dehydrogenase (pLDH) antigen to indicate infection by non-falciparum *Plasmodia* (sensitivity of 95.5%). Previous work has validated rapid diagnostic tests (RDTs) in low-prevalence settings within east Africa, including in Uganda's southwestern highlands, indicating that RDT sensitivity, specificity, and predictive values do not differ appreciably from gold standard polymerase chain reaction (PCR) procedures.^81^–^83^ Here, HRP-II RDTs offered the additional advantage of detecting low-density infections potentially missed by conventional microscopy,^84^,^85^ and their in-field implementation allowed immediate notification to parasite-positive subjects. We referred all individuals with a positive test outcome to staff nurses from Bwindi Community Hospital.

### Statistical analysis.

We estimated parasitemia prevalence by dividing the number of positive test outcomes by the number of tests administered, assuming that all individuals to be at risk for infection. To examine associations between relative risk for positive RDT outcome and individual- and household-level factors within the study population, we built a multivariable log-binomial generalized linear model accounting for demographics (age, sex, and household characteristics), relative nutritional status and food security, behavioral protective measures, knowledge of malaria, and vector exposure through a modified best subsets variable-entry procedure.^86^ This procedure entailed an exhaustive evaluation of combinations of covariables without stepwise selection. The algorithm identified optimal model configurations for set numbers of parameters through information criterion scores.^87^ Tables 1 and 2 indicate the individual- and household-level variables assessed. To prevent overfitting given the small sample size (*N* = 365), we opted *a priori* to consider models with *k* < 10 parameters only. Before model-building, we ruled out collinearity among main effects by Spearman's rank correlation coefficient (ρ) with a cutoff of 10%. We evaluated component plus residual plots to determine appropriate functional forms of continuous and factor variables. After identifying optimal main effect terms, we evaluated potential interactions through a model-based recursive partitioning algorithm testing candidate main effect terms for joint significance.^88^ To avoid model overspecification and compare among fitted models with *k* < 10 parameters, we assessed whether incorporating additional significant and non-significant terms resulted in global model improvement through likelihood ratio tests. Because individuals' risks for infection were stratified by heterogeneous exposure to parasite-positive vectors domestically and within communities, we calculated Huber–White SEs robust to multilevel clustering by household and settlement.^89^,^90^ We considered outcomes with a type I error rate *P* < 0.05 to be statistically significant. We conducted analyses within the R environment (R Foundation for Statistical Computing, Vienna, Austria).

We limited analysis to cases among older children (> 5 years) and adults to avoid confounding caused by two factors. First, cases among individuals over 5 years old are atypical in hyper- and holoendemic areas relative to cases among children under 5 years old.^55^ Lacking longitudinal data on parasitemia within Kanungu in the absence of environmental interventions^60^ or any prior data on malaria among the Batwa elsewhere, we could not assess whether observed prevalence among children 5 years and under during January of 2013 represented typical transmission levels or was affected by exogenous factors of interest to our model; such factors could, however, be assumed to cause a high proportion of infections among individuals over 5 years old in any setting.^1^,^91^ Second, evidence exists for differential treatment-seeking behavior among individuals under and above 5 years of age within east Africa.^92^,^93^ Varied probability for prior malarial diagnosis and antimalarial receipt across ages could bias parasite prevalence estimation in a malariometric survey.

## Results

### Response rate.

Our sampling frame consisted of 630 individuals, among whom 475 individuals were over 5 years old. Of these individuals, we reached 434 individuals, among whom 407 individuals (94%) consented to clinical evaluation, including anthropometric measurement and finger prick. Complete household-level, individual-level, and clinical data were obtained for 365 individuals, representing a 77% overall response rate. Parasite prevalence did not differ significantly between participants who dropped out or completed the full survey (F_1,409_ = 0.01; *P* > 0.9). By Little's test for missingness completely at random, we identified no pattern to data missingness in the variables under consideration (χ^2^ = 59.6; degree of freedom [df] = 53; *P* = 0.25) and expected complete case analysis to yield unbiased results.

### Outcome measures.

Parasite prevalence was 4.1% among participants over 5 years of age and 3.9% among participants of all ages; all parasite-positive cases were attributed to *P. falciparum* caused by HRP-II detection. The adult to child clinical ratio of 3.0 indicated unstable local transmission and generally, an absence of acquired immunity.^55^ We observed a mean MUAC of 24.2 cm (SD = 2.7 cm) among adult males and 23.2 cm (SD = 3.1 cm) among adult females; details of MUAC values by age and sex are available in Table 3. In the 3 months preceding the survey, most (62%) of the sample individuals lived in households experiencing severe food insecurity, whereas fewer individuals experienced moderate food insecurity (32%), mild food insecurity (5%), and food security (< 1%). Age seemed uncorrelated with food insecurity classes (ρ = −0.07). Normal score distributions for MUAC by age–sex class did not differ across food security levels (ρ = −0.08) or between severely and non-severely food-insecure individuals (ρ = −0.06), motivating separate consideration of nutrition status and food security variables. We identified −0.1 < ρ < 0.1 in pairwise comparisons across all candidate covariables presented in Tables 1 and 2, indicating that collinearity among predictors was unlikely.

### Statistical model.

**Figure 2.**
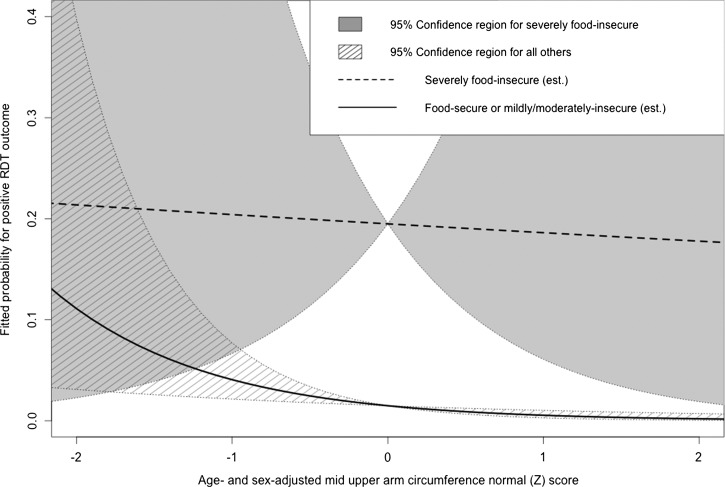
Conditional effects of relative undernourishment by status as severely food-insecure. The fitted probability of a positive RDT outcome is shown for a 25-year-old man who enters the forest regularly and does not own animals or receive outside financial support with respect to relative nutritional status under the scenarios where the man is or is not severely food-insecure. The 95% confidence regions are distinct at *z* scores greater than −1 SD from the mean. Est = estimated.

Table 4 presents the adjusted relative risk (ARR) fitted by the multivariable model as well as the unadjusted relative risk attributed to each factor in univariable regression. Being male (ARR = 3.35; 95% confidence interval [95% CI] = 1.96–5.74), having daily forest exposure (ARR = 1.80; 95% CI = 1.01–3.21), and having severe food insecurity (ARR = 13.09; 95% CI = 2.23–76.79) were significantly associated with elevated odds for positive RDT outcomes. Asset ownership, defined as living in a household that owned an animal or received financial support from family or friends outside the community, was significantly associated with decreased odds for positive RDT (ARR = 0.11; 95% CI = 0.01–0.97). Relative nutritional status, as indicated by within-sample MUAC normal score for age and sex, was significant conditional on household status as food-secure or mildly or moderately insecure. Although the relationship between positive RDT outcome and relative nutritional status was non-significant for severely food-insecure participants (ARR = 2.61, 95% CI = 0.85–7.99), participants who were not severely food-insecure likewise had decreasing odds for positive RDT outcomes with increasing relative nutritional status (ARR = 0.37; 95% CI = 0.19–0.69). Figure 2 depicts this relationship graphically, indicating that the 95% confidence bounds of the fitted probability for parasitemia were non-overlapping between severely food-insecure individuals at most MUAC normal scores.

Variables rejected because of statistical non-significance of additive main effects included sleeping under an insecticide-treated bed net (ITN) the night before the survey, knowledge about malaria and its prevention, time spent in environments other than the forest, knowledge of or reported engagement in domestic and peridomestic malaria prevention efforts, recent travel outside home settlements, and household crowding (measured as the number of children occupying a house). Because only four households reported indoor residual spraying with insecticide within the last 12 months, this variable was not considered. Despite statistical non-significance, retaining age served to reduce noise within component plus residual plots, decrease global deviation (thus, improving goodness of fit), and reduce the largest studentized residuals. Consequently, we force-included age as a continuous variable in candidate models. We identified no significant outlying observations in the model controlling for age (Bonferroni-adjusted *P* > 0.1). An overall χ^2^ deviance test comparing the fitted model with a saturated model with *k* = *n* parameters indicated that deviance for the fitted model was not significantly larger than for the saturated model. Accordingly, we expected a maximally fitted model to offer no significant improvement relative to the present model in accounting for variation in RDT outcomes.

## Discussion

Severe food insecurity may be a risk factor for *P. falciparum* parasitemia among older children and adult Batwa pygmies in Kanungu, Uganda during the biannual high-transmission season. Poor relative acute nutritional status, which was indicated by MUAC *z* score for age and sex, was associated with risk for parasitemia among individuals living in a household that did not experience severe food insecurity in the preceding season. Because we controlled for a protective effect from asset ownership, it is unlikely that severe food insecurity effects owed solely to socioeconomic confounding. Relative undernourishment was not associated with elevated risk for parasitemia among individuals in severely food-insecure households.

The observed 4.1% parasite prevalence in individuals over 5 years old falls within range of prior epidemiologic studies in southwestern Uganda^61^ and above the lower measurement reliability limit of malariometric surveys.^91^,^94^ Reference MUAC measurements among ethnically similar Twa group pygmies are available from surveys conducted in the early 1980s among forest-dwellers in the Democratic Republic of the Congo; these measurements indicated 23.3 cm mean MUAC among adult males in the Kivu region bordering Kanungu during an unspecified season^95^ and 22.6 cm mean MUAC among adult females after the rainy season, similar to the timing of our survey.^96^ These historical measurements fell roughly within the second quartiles of observed distributions in our survey (Table 3). Such marginal gains in MUAC among the Batwa evidenced little progress in nutritional success, despite rising life expectancies in the Ugandan and Congolese general populations over the intervening decades.^97^

Normal scores from observed MUAC distributions within the sample here allowed us to assess how risk for positive RDT outcome varied among Batwa individuals of differing acute nutritional status. Using international standards derived from non-pygmy populations for MUAC, cutoffs would likely have led to overestimating malnutrition prevalence within our sample, because growth trajectories differ between pygmies and non-pygmies^98^; among pygmies, average MUAC is lower than international standards across sexes and all ages greater than 5 years old.^70^,^71^ Without knowing how Batwa ethnicity and inadequate nutrient intake respectively contributed to individuals' MUACs, we lacked a basis for interpreting observed MUAC measurements in the context of international cutoff values and accordingly, identifying wasting in an absolute sense.

In previous parasitological surveys, associations between malnutrition and risk for parasitemia seemed to differ from those associations involving risk for clinical illness. A study from a *P. falciparum*-endemic region of Colombia suggested elevated risk for parasitemia in acutely undernourished individuals, showing log-linear increases in parasite density with decreasing BMI.^99^ Cross-sectional surveys in Kenya identified associations between parasitemia and chronic undernutrition among children under 36 months but did not identify significant associations between parasitemia and wasting.^100^ Clinical trials in Uganda have additionally shown elevated risk for recurrent parasitemia after clearance in chronically undernourished children (however, without corresponding risk elevation among the acutely undernourished).^101^

As measured here, a household's experience of food insecurity involved reducing portion sizes or skipping meals, reducing dietary quality and diversity, and worrying about running out of food in the 3 months before the survey.^65^ The metric differentiated severely food-insecure from other food-insecure households according to experienced hunger rather than reduced dietary quality and diversity, which had greater relevance for distinguishing between food-secure and -insecure households. Experiencing acute hunger indicated that individuals in severely food-insecure households likely lacked sufficient caloric intake and may have been at risk for protein-energy malnutrition^102^; given the observed prevalence of lesser food-insecure states, nearly all individuals surveyed were likely at risk for micronutrient deficiencies associated with low-quality, low-variety diet.^103^

Elsewhere in Uganda, household- and individual-level risk factors associated with low MUAC have been shown to differ from those factors associated with other malnutrition outcomes.^98^ Accordingly, an interaction effect between MUAC and food insecurity status may indicate that living in a severely food-insecure household and having low MUAC here acted as a proxy for differing nutritional risks and physiological outcomes and had distinct causal implications for risk for parasitemia. Because of its sensitivity to decreasing fat or muscle stores, low MUAC may have acted as a measure of near-term inadequate nutritional intake, conveying effects of acute undernutrition on susceptibility to *P. falciparum*.^37^,^80^,^99^,^104^,^105^ We do not know if households' food-insecurity experiences recalled during the 3 months before surveying were exceptional, seasonally cyclic, or chronic in nature. In the event that reported experiences were not atypical for Batwa households, the severe food-insecurity variable may have captured risk for parasitemia effects associated with chronic undernutrition. Conditional significance may indicate that increased risk for parasitemia associated with chronic undernutrition (for instance, because of severe food insecurity) outweighed effects owing to acute undernutrition, which was suggested in other cross-sectional parasitological studies.^100^,^101^ Alternatively, low MUAC for age and sex may have been attributed to wasting from chronic medical conditions, including malarial anemia or undiagnosed human immunodeficiency virus (HIV) infection^106^; although a cohort study among non-Batwa persons in the region indicated 7.7% HIV-1 seroprevalence in 2005,^107^ only one person within our survey self-reported HIV-positive serostatus and receipt of antiretroviral treatment. Contributions of unobserved factors, including HIV, to MUAC reductions would presumably be less statistically apparent in individuals from severely food-insecure households than the rest of the sample, because food shortages would likely cause a high relative proportion of all-cause wasting in the most severely food-insecure households.

In the event that downstream physiological effects of severely constrained food access may have been similar to those effects seen among persons with low MUAC,^108^,^109^ the binary severe food-insecurity variable may have served as a catchall variable representing such immune effects. Small sample size and rareness of positive RDT outcomes indicate that our study may be at risk for a spurious finding of this nature. In such an event, variation in MUAC may have achieved conditional significance, because it contributed meaningfully to the model only among individuals in households for which the severe food insecurity indicator did not apply.

Potential protective effects of iron and folic acid deficiencies against clinical illness confound implications of a malnutrition–parasitemia risk association for reducing malaria morbidity and mortality. A recent meta-analysis^31^ indicated that iron and iron plus folic acid supplementation may be unsafe in areas of intense malaria transmission unless accompanied by expansion of disease surveillance and treatment services. Likewise, the World Health Organization has advised against implementing such supplementation in malaria-endemic settings.^110^ Certain micronutrient combinations seem *in vitro* to bolster the cytokine-mediated immune response to *Plasmodia*,^111^ and supplementation with zinc, vitamin A, or both has proven varyingly efficacious against clinical illness in field trials.^112^–^118^ These trials have nonetheless produced minimal evidence that supplementation protects against parasitemia.

Individual exposure-related variables may have had particular significance in the present study because of malaria ecology and transmission dynamics in Kanungu. Low parasite prevalence, a lack of parasitological evidence for premunition, and a high adult to child case ratio here suggested that naturally acquired immunity was negligible within the population.^13^ In hyper- and holoendemic settings, where exposures are ubiquitous and most often indoors, young children have elevated risk for *P. falciparum* infection because of their lack of acquired immunity, whereas men and non-pregnant women typically do not differ in risk for infection.^119^ Elevated risk among men and among individuals who regularly entered the forest here suggested outdoor vector exposures, particularly in recently deforested landscapes, where microclimatic factors may have led to elevated mosquito vectorial capacity.^120^ Elevated risk among men and persons with outdoor exposures typically indicates that malaria is an occupational disease in populations lacking premunition, where risk relates to sexual division of labor.^16^,^17^ Non-significant protection from sleeping under ITNs (relative risk [RR]= 0.66; 95% CI = 0.34–1.25), moreover, suggested that transmission was unlikely to occur indoors.^121^ A significant effect from crowding, which was indicated by the number of children within a household, would likewise typically suggest domestic or peridomestic exposure^18^; here, this variable was non-significant (univariable RR = 1.13; 95% CI = 0.82–1.55). Although unknown vector distribution in the study region prevented us from interpreting these outcomes in the context of *Anopheles* ecology, recent work elsewhere within the east African highlands suggests a shift to exophagic and exophilic species compositions relative to historical observations.^122^,^123^ Throughout Africa, such changes to vector ecology have challenged domicile-based interventions, such as ITN distribution and indoor residual spraying.^18^,^124^

Our cross-sectional study design did not facilitate inference into the causal nature of observed nutrition and food security associations with *P. falciparum* parasitemia. Reductions in MUAC associated with acute malnutrition, for instance, may have occurred subsequent to infection, particularly if coaffected individuals suffered from chronic parasitemia or malarial anemia. In the latter event, reverse causality could dictate that infection, in fact, contributed to low MUAC. Although associations with food insecurity and relative undernourishment may suggest host nutrition as a factor in risk for parasitemia, our lack of information about associations with specific nutrient deficiencies limited mechanistic inference in this relationship. Also, our sample was small and included only 15 positive RDT outcomes. We lacked statistical power to reliably estimate small effect sizes, for instance, from sleeping under ITNs, and such findings may have been subject to type II error. High prevalence of severe food insecurity likewise indicated that our outcomes may have lacked specificity; although psychometric properties of the instrument are unlikely to reduce its validity in underdeveloped and resource-poor contexts,^67^,^125^,^126^ the predominance of severe food insecurity among observed states indicated the population's food-insecurity experiences were among the most severe that the USDA survey metric could identify. Taken together with the Batwa's exceptionally low life expectancy and historic socioeconomic marginality, they may indicate that outcomes from the present study are not sufficiently generalizable for informing regional intervention priorities.

Our findings motivate future longitudinal studies suited to identifying temporal and causal associations among the variables assessed here, which may account for immunological factors underlying nutritional associations with parasitemia. Two important objectives for understanding present outcomes will be (1) characterizing whether risk for food insecurity varies in phase with risk for *P. falciparum* infection, particularly in settings where both oscillate intra-annually, and (2) establishing whether malnutrition-associated wasting precedes or follows infection among individuals who are not severely food-secure. Characterizing livelihoods among individuals who are severely food-insecure may likewise identify behaviors or exposures associated with risk for *P. falciparum*, accounting for non-nutritional factors explaining why persons in severely food-insecure households here had elevated risk for infection. Such research could extend current evidence regarding the safety or suitability of food security and nutritional interventions in *P. falciparum*-endemic settings and address differential implications of supplementation for parasitemia and malaria morbidity.

## Figures and Tables

**Table 1:** Characteristics of the study population (individuals)

Variables	Total *n* (%)	Males *n* (%)	Females *n* (%)
Age (years)	365 (100)	167 (100)	198 (100)
6–12	103 (28)	53 (32)	50 (25)
13–23	92 (25)	42 (25)	50 (25)
24–35	81 (22)	30 (18)	51 (26)
36–47	40 (11)	20 (12)	20 (10)
48–59	29 (8)	13 (8)	16 (8)
60+	20 (5)	9 (5)	11 (6)
Malaria infection	15 (4)	11 (7)	4 (2)
Protective measures			
Sleeping under ITNs last night	174 (48)	82 (49)	92 (47)
Environment (clearing brush near home, draining stagnant water)	143 (39)	69 (41)	74 (37)
Closing windows and doors at night	125 (34)	57 (34)	68 (34)
Daily forest exposure			
Spends no time in forest during the day	189 (53)	89 (53)	106 (54)
Spends less than one-half of the day in forest	150 (41)	64 (38)	86 (43)
Spends about one-half of the day in forest	14 (4)	6 (4)	8 (4)
Spends more than one-half of the day in forest	3 (1)	3 (2)	0 (0)
Spends all day in forest	6 (2)	6 (4)	0 (0)
Spends time at night in forest (not excluding other categories)	4 (1)	0 (0)	4 (2)
Other daily environmental exposures			
Any time in fields	265 (73)	112 (67)	153 (77)
Any time near rivers or lakes	236 (65)	104 (62)	132 (67)
Any time in or near animal sheds	58 (16)	26 (16)	32 (16)
MUAC			
≤ 1 SD below mean for age and sex	46 (13)	22 (13)	24 (12)
−0.99–0 SD below mean for age and sex	152 (42)	67 (40)	85 (43)
0.01–1 SD above mean for age and sex	116 (32)	51 (31)	65 (33)
> 1 SD above mean for age and sex	51 (14)	27 (16)	24 (12)
Knowledge of malaria			
Identifying mosquito as vector	290 (79)	140 (84)	150 (76)
Identifying ITNs as a preventative measure	155 (43)	73 (44)	82 (41)
Identifying insecticide sprays as a preventative measure	88 (24)	38 (23)	50 (25)

**Table 2:** Characteristics of the study population (households)

Variables	Households *n* (%)	Individuals *n* (%)
	120 (100)	365 (100)
Malaria infection	13 (11)	15 (4)
Household food insecurity		
Food security	1 (< 1)	1 (< 1)
Mild food insecurity	8 (7)	19 (5)
Moderate food insecurity	44 (37)	117 (32)
Severe food insecurity	67 (56)	228 (62)
Protective measures		
Living in a household with indoor residual spraying in past 12 months	4 (3)	18 (5)
Household wealth indicators		
Household owns any livestock	27 (23)	87 (24)
Household receives money from family/friends outside the community	23 (19)	84 (23)
Either household wealth indicator	45 (38)	146 (40)
Number of children living in household		
0–1	40 (33)	79 (22)
2–3	37 (31)	97 (27)
4–5	35 (29)	137 (38)
6–7	8 (7)	52 (14)

**Table 3:** MUAC observations

Age/sex	Percentile	MUAC (cm)	MUAC normal score
6–8 years			
Male	25	15	−0.89
Male	50	17	0.08
Male	75	18	0.57
Female	25	16	−0.55
Female	50	16	−0.55
Female	75	18	0.33
9–11 years			
Male	25	16	−0.78
Male	50	17	−0.25
Male	75	18	0.28
Female	25	16.3	−0.50
Female	50	17	−0.24
Female	75	18.8	0.38
12–14 years			
Male	25	17	−0.69
Male	50	18	−0.27
Male	75	20	0.56
Female	25	20	−0.53
Female	50	20.5	−0.38
Female	75	22.8	0.33
15–17 years			
Male	25	19.3	−0.85
Male	50	22.5	0.29
Male	75	23	0.46
Female	25	22.8	−0.57
Female	50	23.5	−0.32
Female	75	27.3	0.89
18 + years			
Male	25	22	−0.83
Male	50	24.3	−0.08
Male	75	26	0.68
Female	25	21	−0.71
Female	50	23	−0.07
Female	75	25	0.57

**Table 4:** Results of the log-binomial generalized linear model

	RR (95% CI)	
	Univariable (unadjusted) RR	Multivariable ARR
Age (years)	1.01 (0.97–1.05)	1.01 (0.97–1.04)
Male sex	3.26* (2.02–5.27)	3.35* (1.96–5.74)
Daily forest exposure	2.20 (0.87–5.53)	1.80† (1.01–3.21)
Asset ownership	0.11† (0.01–0.98)	0.11† (0.01–0.97)
Severe food insecurity	8.41‡ (1.77–39.98)	13.09‡ (2.23–76.79)
Age/sex-adjusted MUAC *z* score	0.83 (0.43–1.61)	
Not severely food-insecure		0.37‡ (0.19–0.69)
Severely food-insecure		2.61 (0.85–7.99)

* *P* < 0.001.

† *P* < 0.05.

‡ *P* < 0.01.
